# May I have your uterus? The contribution of considering complexities preceding live uterus transplantation

**DOI:** 10.1136/medhum-2020-011864

**Published:** 2021-02-24

**Authors:** Lisa Guntram

**Affiliations:** TEMA—Department of Thematic Studies, Linkopings universitet Institutionen for Tema, Linkoping, Sweden

**Keywords:** gender studies, medical humanities, social science, reproductive medicine, medical ethics/bioethics

## Abstract

Uterus transplantation combined with in vitro fertilisation (IVF) (henceforth called UTx-IVF) as a treatment for infertility caused by an absence or malfunction of the uterus is advancing. About 50 transplantations have been conducted worldwide and at least 14 children have been born—9 of them by women taking part in a Swedish research project on UTx-IVF. The Swedish research protocol initially stated that the potential recipient must ‘have her own donor’ who is preferably related to the recipient. But what does it mean to ask someone for a uterus? What challenges does this question instigate? And what norms may it enact? In this article, I explore how 10 women—who have considered, and sometimes pursued, UTx-IVF—describe their experiences of searching for a donor. I aim to show how an analysis of such accounts can help us unpack some of the specific relational and gendered dimensions of UTx-IVF and by doing so enrich discussions of risks, benefits, care and support in UTx-IVF. Drawing on research in social sciences and medical humanities that has demonstrated how assisted reproductive technologies and organ donation can provoke social and familial conundrums, with respect to such topics as embodiment and identity, I present three patterns that describe different dimensions of the interviewees’ quest for a uterus donor. I discuss the negotiations that took place, how expectations unfolded and how entanglements were managed as the interviewees considered asking someone for a donation. Such an examination, I suggest, contributes to make care and support more attuned to the experiences and entanglements that UTx-IVF entails for those pursuing it. This will become increasingly important if (or when) UTx-IVF becomes part of general healthcare. To conclude, I problematise responsibilities and relational challenges in medical innovation, and in this way provide insights into how the ethical debate over UTx-IVF can broaden its scope.

## Introduction

During the past 20 years, research in uterus transplantation has advanced rapidly. Since the late 1990s, Swedish researchers have systematically investigated the possibility of uterus transplantation combined with in vitro fertilisation (IVF) (henceforth called UTx-IVF), at the forefront of endeavours to find a medical cure for uterine-factor infertility (UFI).1, 2 By July 2019, 14 children worldwide had been reported to have been born as a result of UTx-IVF, 9 of them by women taking part in a Swedish research project.3

In UTx-IVF, organ transplantation intersects with assisted reproductive technology (ART) as the procedure aims to enable gestational and—in cases so far—genetic motherhood. As such, it is—in contrast to several other forms of organ transplantation—not vital for the recipient but falls within the category of life-enhancing transplantations. Furthermore, UTx-IVF is temporary as the uterus will be removed after a determined period of time.4 In mother-to-daughter donation, UTx-IVF also involves the recipient receiving the uterus she herself was carried in.5 These specificities of UTx-IVF bring potentially new dimensions of embodiment and relationality into discussions of complexities in live related organ donation and connect them to investigations of how ART alters reproductive choice, kinship, agency and female solidarity.

In this article, I explore 10 Swedish women’s accounts of considering, and in some cases pursing, UTx-IVF. The Swedish research project on UTx-IVF initially involved live donors6 and required that a potential uterus recipient must ‘have her own donor’.7 It was specified that it is preferable that the donor be the ‘recipient’s mother, maternal or paternal aunt or older sister’, while making it clear that it may become possible in the future to use a uterus from an unrelated, so-called altruistic, donor.8 But what does it take to ask someone for a uterus? What challenges does this question instigate? And what norms may it enact?

Taking these questions as my starting point, I examine in particular how the interviewees describe their experiences of searching for a donor. By investigating the considerations and concerns that precede UTx-IVF from the perspectives of potential recipients, I aim to show how an analysis of live related uterus donation can help us unpack some of the specific relational and gendered dimensions of UTx-IVF. In doing so, I also seek to enrich discussions of risks, benefits, care and support in ethical debates, and the clinical settings of UTx-IVF.

The advancements in UTx-IVF have initiated debates concerning live versus deceased donors9; priority-setting and access to treatment10; regulatory difficulties and reproductive rights11 and social expectations and norms concerning parenthood and gender.12 However, analysis of the experiences of those potentially in need of UTx-IVF and their perspectives on the procedure are scarce, and while the need for such knowledge has occasionally been acknowledged,13 it remains absent in the general ethical discussion of UTx-IVF and in the particular discussions of risks and benefits. In such discussions, benefits for the recipient have been suggested to include the value of becoming a gestational, genetic and social mother.14 Discussions of risks for the recipient have addressed the surgeries involved (transplantation, caesarean section, hysterectomy), immunosuppressive treatment and pre-eclampsia and occasionally also emotional aspects, such as the risk that UTx-IVF nurtures the therapeutic misconception, misjudgement of risks and wishful thinking.15, 16 Benefits for donors have been suggested to include the opportunity to alleviate the suffering of an individual who is involuntarily childless, and—in the case of donation between family members or friends—the opportunity to assist a loved one.17 Discussions of risks for the donor have focused on the physical risks of hysterectomy, and especially the long duration of the procedure.18 However, while psychological effects of hysterectomy have occasionally been addressed in previous research on UTx-IVF,19 knowledge of women’s lived experiences of undergoing hysterectomy is, in general, limited20, 21 and rarely addressed in discussions of UTx-IVF.

In this article, I seek to contribute to filling a part of the persisting knowledge gap with respect to the experiences of women who are positioned as or who finds themselves to be ‘in need’ of a uterus. By analysing accounts of women considering UT-IVF, it contributes with perspectives from women who find themselves, or who are positioned as, being ‘in need’ of a uterus. As such, the article enriches our understanding of social expectations on motherhood and of gendered dimensions in UTx-IVF22 and contribute to nuance discussions about live versus deceased donors.23 Drawing on the rich scholarship on organ transplantation and ART in medical humanities, feminist studies and the social sciences, I present in what follows three patterns that together form an overarching storyline of the core considerations and entanglements at stake in the interviewees’ quest for a uterus donor. In conclusion, I discuss how these patterns add nuances to the ethical debates and clinical practice of UTx-IVF.

### Complexities in the intersection between ART and organ transplantation

In recent decades, ART and organ transplantation alike have become ordinary parts of healthcare. At the same time, these developments bring new questions about their potentially unsettling and far-reaching consequences, questions that are relevant also to UTx-IVF, as this form of transplantation specifically aims to enable the birth of a child.

Feminist and anthropological scholars have analysed the ways in which ART renders visible, and at times challenges, beliefs about kinship, and how it enables new forms of reproductive choice, agency and new forms of female solidarity. They have, at the same time, demonstrated how ART on the one hand may foster new forms of domination, control and subordination—for example, through the trafficking in human organs and bodily services—and on the other hand may foster resistance.24 As such, ART offers reproductive potential but, as Bob Simpson25 notes, it ‘also brings in relationships and social knowledge that are unintended or unwanted and, perforce, must be absorbed, modiﬁed or negated’. The expanded choices in the field of ART, argues Susan McKinnon, thus come with an ‘extraordinary amount of work’ which ‘calls into question the naturalness of the otherwise seemingly “natural occurrence” of procreation—and, more importantly, of nature, itself’.26

Similarly, live organ donation calls for relational negotiations as it involves a non-therapeutic, risky procedure being performed on a healthy individual (the donor), who—in addition—often has a close relationship with the recipient (as parent, child or sibling).27 Furthermore, organ donors, Nancy Scheper-Hughes argues, are often ‘responding to family pressures and to a call to “sacrifice”’.28 For some, the experience of donating may indeed be positive, yet decisions about giving or accepting an organ are shaped by expectations on social roles and on the donor-recipient relationship that are figuring in the societal and interpersonal context in which the donor-recipient dyad is situated.29 As live organ donation poses a risk to the health, well-being, and day-to-day life of the donor, an offer of donation may be perceived as emotionally burdensome by prospective recipients and donors alike.30 For example, in live kidney donation from parent to child, parents may express their willingness to donate and describe donation as ‘obvious’, while at the same time stating that they find themselves unable to say ‘No’ when approached.31 Prospective organ recipients may, moreover, state that they find it difficult to ask for a donation, while at the same time expressing a wish to take responsibility for asking.32 However, knowledge-gaps remain with respect to prospective recipients’ perspectives on relational constraints and pretransplantation experiences.33 Fear of disappointing the donor if the organ would not function properly may, for example, as suggested by de Groot *et al*, be perceived as having an impact on the relationship by the recipient but go unnoticed or unrecognised in studies of donors’ perspectives.34

Focusing on the embeddedness of economic markets Michel Callon expands on the complex relationships in organ donation as he uses the case of postmortem organ transfer to describe the concepts of entanglement and disentanglement.35 While money circulates freely and anonymously in markets and as such stay disentangled, the exchange of whole organs is, Boers *et al* state as they draw on Callon, ‘profoundly involved in kinship, mortality, bodily relations and immunological relations’.36 Money, Cathrine Waldby and Robert Mitchell furthermore suggest, serves to ‘disentangle objects from their owners by providing equivalence’.37 Buyers and sellers do ultimately not remain in a relationship when the transaction is completed. The circulation of a solid organ stands in stark contrast to such monetary transactions as they ‘circulate in the most personal and time-limited form’.38 How is it then possible, Callon39 asks, to circulate an organ:

between a donor—generally dead—and a recipient—generally in danger of death—when the organ is entangled in the body of a potential donor and through him in his family or circle of friends?

Organ transfer requires, Callon argues, successful disentanglement. However, this is not easy. It requires, that the organ is ‘transformed into a good free of all attachments’, which—in turn—necessitates that the transfer takes the form of a gift that ‘reconciles circulation and entanglement’. In some contexts, Callon suggests with reference to the work of Fox and Swazey, concerted and systematic efforts have been made to disentangle organs by attempting to transform them ‘in something which makes them more like goods than gifts’. However, Callon continues, the more investments made to disentangle an organ from a donor, for example, by listing its relations of attachment, the ‘more the ties proliferate and multiply’.40 In line with others’ work on relation complexities in organ donation, Callon41 suggests however that disentanglement can always be only partial.42 Discussing the concept of ‘sharing’ as a way of understanding bodily exchanges in medicine (such as the donation and transfer of blood, eggs, sperm and organs), Kristin Zeiler further demonstrates the partiality of disentanglement. Sister-to-sister egg donation, and the extraction of ovas from the donor’s body, both involve the ‘managing rather than the cutting of social, genetic and emotional ties between the eggs, the donors and their bodies, and other family members’.43

Much work in medical humanities and social sciences relatedly demonstrates the complexities of organ donation. And, as Scheper-Hughes44 argues, since the relational and emotional contingencies that emerge when an organ is moved from one body to another are less visible than potential medical complications, there is a risk that such contingencies fall outside the view of transplant professionals. To consider and understand them requires, therefore, research approaches and research skills that can capture the social and familial conundrums associated with live donation. For example, through examinations of how transplantation may disturb embodiment and personal identity, it becomes possible to understand in more depth adverse outcomes, and improve the consent processes, preoperative teaching and follow-up care.45 I contribute to this endeavour by using Callon’s concepts of entanglement and disentanglement as tools in an investigation of how relational complexities preceding UTx-IVF are managed.46

## A thematic approach: interviewees, material and analysis

The material analysed in this article consists of interviews with 10 women, 26–37 years of age, who had discovered in their teens that they did not have a uterus. Six of them took part in one interview. Out of the remaining four interviewees, three took part in three interviews and one of them took part in two interviews. These women were all being evaluated for transplantation at the time of the first interview or shortly thereafter. This gave me the opportunity to follow their transplantation journey for a longer period.

The interviewees were all Swedish citizens and they all presented themselves as heterosexual. At the time of the interviews, some of the interviewees had only just started to consider transplantation; some were being evaluated for eligibility for transplantation, and some had undergone transplantation. All of them had considered the possibility of adopting and/or arranging for a surrogate mother abroad. In general, they described adoption as a time-consuming process fraught with uncertainties, while their descriptions of surrogate arrangements centred on the high cost. They also described the difficulties of ensuring that an arrangement would feel morally acceptable. These descriptions of alternative routes to parenthood need to be understood in light of Swedish regulations of surrogacy and adoption practices. Although surrogacy is not specifically regulated in Swedish law, it is in practice forbidden since only single women and couples who include a woman who can carry and give birth to an intended child can access fertility treatment.47 With respect to adoption, intercountry and national adoptions take place. However, intercountry adoptions are far more common. In 2013, 350 intercountry adoptions of children were carried out in Sweden in comparison with 45 national adoptions of children not related to the adoptive parent(s).48

The interviewees were recruited in several ways: following distribution of advertisements in networks that I had previously established with women living with a congenital absence of the uterus and the vagina; through my professional website at the university; in social media; in networks of which women with UFI were members and by contacting directly women with UFI who had shared stories about their lives in traditional or social media. The advertisement stated that the aim of the project was to explore how UTx-IVF was experienced by women who had undergone or were considering UTx-IVF, their expectations on the procedure and their reflections on embodiment and the risks and emotions in relation to UTx-IVF. It also specified that I wanted to obtain women’s experiences and perspectives in their own words, and included information on what participation involved.

The 10 women recruited were informed that they could end their participation at any time without having to explain their reasons for doing so; that collected data would be treated confidentially, and that documents with identifying information, such as consent forms, were to be kept in secure conditions at (information removed during review). They were also informed that they would receive a copy of any scientific publications, along with a summary of the same. They all gave their informed consent to participate. The interviews used a semi-structured interview-guide organised around overarching themes. The questions were open-ended and intended to enable the interviewees to describe their experiences and to provide a context that allowed a conversation to take place between me as the interviewer and the interviewee. Each of the interviews covered all of the themes in the interview guide, but since the interviewees were encouraged to tell their story the order of the questions differed. Furthermore, the specific questions were refined and reformulated in light of insights gained from the interviews.

The interviews took place at a location chosen by the interviewee; two preferred to meet in my office, three in a private room at a public library and five in their homes. During the interviews, I asked the interviewees to describe what it was like to live with UFI, their experiences of and/or thoughts on UTx-IVF and their expectations for the future. In particular, I tried to facilitate the interviewees’ narrations of UTx-IVF in light of their embodied histories of living without uterus and vagina and the embodied dimensions of their relations to family and friends.

When planning and conducting the interviews, I strived to stay reflexive and sensitive to how my position as a researcher—its situatedness and embodied particularities—always impacts the relationship between my interviewee and myself as a researcher.49 I wanted to let the interviewees know about my research interest and their grounds, while at the same time avoiding claims about being able to fully grasp the complexity and nuance of their embodied experiences and histories. I also considered the meaning of my own embodiment in the research situation, for example, reflecting on the meaning of my gender, age and ethnicity, and specifically on what it would mean if I had been pregnant when conducting the interviews. I benefited here from my previous experience in researching topics that can be considered, or can become, sensitive to the interviewees.50 This allowed me to take into account conditions that contribute to creating an interview situation in which the interviewees feel comfortable sharing their stories.

I conducted all interviews in Swedish and they were all audio-recorded. Qualitative research has underscored the importance of also considering the situatedness and interpretative elements of transcription.51 In this study, I transcribed all recordings myself word by word. During this process, I initiated my analysis of the material, for example by paying specific attention instances where the interviewees expressed emotions and marking nuances such as crying and laughter. This enabled me to stay sensitive to emotions expressed in the interviewees’ accounts of their embodied and relational experiences of, expectations on, and concerns with UTx-IVF.

I subsequently used an inductive thematic approach to analyse the considerations and concerns that my interviewees described as preceding UTx-IVF.52 In doing so, I did not search for preset themes but identified patterns in the material. Furthermore, I viewed the interviewees’ accounts as bundles of different kinds of descriptions of previous experiences and description of the future, through which individuals make sense of UTx-IVF. They may, at times, be centred on a certain event, or they may focus more broadly on experiences that are meaningful to the interviewee.53 Using the analytic software Atlas.ti, I first coded each interview by paying specific attention to the interviewees’ descriptions of what they cared about, in a broad sense, when it came to UTx-IVF. I then compared recurring ways of narrating the search for a donor, and in this way identified matters of concern that spanned across several interviews. I also addressed how the interviewees made sense of these matters in their specific circumstances.54 Throughout this process, I strived to stay reflexive about the inherently embodied dimensions of analysis, including also the relationship between the interviewees and myself and the positioning of the interviewee and the interviewer that this relationship involves.55 Given that I was the single coder of the material I presented my preliminary findings both at the work-in-progress seminar series BoKS: Body, Knowledge, Subjectivity, Department of Thematic Studies, Linköping University and at the workshop 'Re-Imagining Transplantation: The Politics andPoetics of Embodiment and Identity' arranged by the Nordic Network Gender, Body, Health. On both occasions, I received valuable feedback and constructive questions to develop my coding and analysis.

In the analysis, I identified three patterns that together formed an overarching storyline of the core considerations and entanglements at stake in the women’s search for a uterus donor. In my presentation of these patterns below all the quotations have been translated by me and all names have been replaced by pseudonyms.

### Patient and public involvement statement

The research questions that guided the research project of which this article is part developed from my previous research on women’s experiences of living with a congenital absence of vagina and uterus.56 As such, the overall project drew indirectly on perspectives of affected individuals and involved affected individuals explicitly through its use of qualitative interviews as its method for data collection. Furthermore, the qualitative research design of the project, which had been approved by the regional ethics board, led to the aims and research questions being continuously reconsidered and developed in light of the gathered data. In this way, the research questions were guided by the experiences, expectations and preferences with respect to UTx-IVF of those affected. Results from the project will be disseminated through a broad range of channels: in press releases from the university; through social media (such as my research blog and the social media channels used by my department); networks that gather women with UFI; in Swedish on a Swedish Wikipedia page on UTx-IVF (to be created) that will include perspectives from medical humanities and on the existing English Wikipedia page on UTx-IVF.

## The quest for a donor

The fact that it was necessary to bring your own donor when looking to participate in the Swedish research project on UTx-IVF was well-known to most of the interviewees. Most of them had considered this requirement long before they actively began to investigate the possibility of having a transplantation. But whom do you ask for a donation? What must be considered?

The first pattern formed around the ways in which the interviewees told of how they initially approached their mother in their search for a uterus. In most cases, the interviewees said that their mother stated that she would obviously donate, if medically allowed to. This pattern, I suggest, teased out relational expectations that play out when faced with the task of finding a uterus donor, and the challenges that arise when such expectations are not met.

The second pattern detailed the interviewees’ consideration of whom to ask when one’s mother could not, or would not, donate. This pattern spelled out expectations on women’s life trajectories and reproductive bodies in general, and described anticipated relational contingencies. In this way, it demonstrated how the question of donation is not always confined to the one asking and the one being asked, but can become a concern that is distributed across the relational entanglements of the prospective recipients and donors.

The third pattern concerned accounts that described what had happened when the quest for a donor came to an end in cases in which the mother could not or would not act as donor. It told of experiences of unexpectedly being offered a uterus and moving on to transplantation; of the potential donor not being medically accepted for donation and of not finding a donor. Specifically, this pattern detailed the ways in which the interviewees had continued to search for a donor, and ultimately how they had (or tried to) move on.

Together, the three identified patterns form an overarching storyline of core considerations and entanglements at stake in the women’s quest for a uterus donor.

### Asking mum? Expected and disrupted entanglements

I’d be happy to, why not? …]57 You are my beloved daughter.

The quotation above, from Eva about her mother’s response when asked about donation, is typical: most of the interviewees told of how their mothers had stated that it was obvious to say ‘Yes’ when asked about donating their uterus. While the interviewees pondered on the risks, most of them said that their mother seemed sure that they wanted to donate, a pattern also seen among parents who are asked to donate a kidney.58 Agnes, for example, said that her mother had been very assertive about her willingness to donate.

Because of course you think about that [the risks] too, about mom and all that. But mom was so sure about this the entire time. She just said: “I’ll do it, 100%. You don’t even have to think about it”, you know. She just said: “You don’t even have to think about my risks, because I’ve already thought about them, *you know*.59

Most of the interviewees also said that, despite the risks, it felt obvious for them to ask their mother. In many cases, they stressed that they had talked to their mother about the possibility of donation when they had found out that they did not have a uterus, and long before investigating transplantation. However, many also stressed that it did not feel important to obtain a uterus from their mother as such. One of them, Emma, had not seriously investigated the possibility of UTx-IVF when we met the first time, but saw it as a future possibility.

Well, my mother said very early on that “I’ll do this”, you know. So I feel that I’ve always known that there is an alternative. But I don’t think… it’s not… If you think about it only in theory, I don’t think it matters that much. Because it is not something that you keep afterward. As I’ve understood it. They will take it out. When *you are done*, you know. So it feels more as a… as means for it to work out well, rather than a question of it feeling right.

In order to transfer a uterus from one body to another it needs—to use Callon’s conceptualisation—to be physically disentangled from the body of the donor and then re-entangled in the body of the recipient. However, before the disentanglement of the uterus from the donor’s body can take place, it must have been agreed that the uterus will be disentangled a second time—from the body of the recipient. This ephemeral60 feature of UTx-IVF was what Emma drew on as she considered whom to approach for a donation. Since the uterus would not stay in her body for life, but would be removed within a certain period and after a predefined number of pregnancies, it became not primarily a matter of it *feeling* right, but a matter of *feasibility*—of simply making it all ‘work out well’. For her, the knowledge of future disentanglement decreased the importance of who would become her donor. Her story suggested that this however might be something that she would consider more carefully if future disentanglement was not required. Furthermore, Emma stressed that since her mother had offered to donate early on, she had always known that this was an alternative. Hence, the proximity of a potential donor provided comfort and reassurance of a future in which her infertility could be managed.

Occasionally, the question of a donation illuminated more explicit expectations on the mother-daughter relationship. Such expectations were most apparent in Melinda’s story of her mother’s response when approached with the question of donation. Melinda described how she had hoped that her mother would donate, but her mother had declared quite early on that she would not want to. It was at this point, Melinda emphasised, that ‘one of the greatest rifts between us started to grow’. Melinda felt, she explained, that her mother had not ‘taken on that “motherly” responsibility’ when Melinda was growing up.

And when she said that [that she would not donate], then I realized that: “Shit, where is the love between child [sic]… and daughter?”. Because if I had had a child then I would have given a kidney, anything. You would do anything for your child.

When her mother considered things like cosmetic results and menopause, Melinda felt ‘Well, she can’t have the right kind of love for her child”. Her mother’s response had fostered an embitterment in Melinda and even if her mother since then had asked for forgiveness and offered to donate, Melinda was still sure about her stance. ‘I don’t want anything from her!’ she underscored, adding that it would feel a lot better to have a uterus from a deceased donor. Melinda had been told to let go of that thought and to be grateful, but she was adamant; ‘I don’t want anything from her inside of me’.

Indeed, the way in which Melinda described her own reaction to her mother’s response was an exception in my material. Interviewees in general stated that they understood any hesitation from their mother and emphasised that their mother had been more assertive about wanting to donate than the interviewee about receiving. Nevertheless, I find Melinda’s story to be important because it illustrates what failed expectations can lead to. In Melinda’s case, the mother-daughter relationship was expected to constitute a relational entanglement in which (physical) disentanglement (from the uterus on her mother’s part) was taken for granted. When this expectation was not met, it resulted, as Melinda put it, in a ‘rift’ and an ‘embitterment’, which made a relational re-entanglement seem impossible and made a future physical entanglement through transplantation seem unthinkable.

The ‘obviousness’ of mother-to-daughter donation that accounts in the first pattern expressed could, at first glance, be seen as shaped by two facts: that it was required that you bring your own, preferably related, donor when seeking to take part in the research on UTx-IVF in the Swedish context, and that your donor should have had successful pregnancies and not want any more children. In light of these requirements, which limited from the onset the recipients’ choices and options one’s mother may stand out as one’s primary donor candidate when searching for a donor in the Swedish context. However, several of the interviewees said that they had talked with their mothers about donation long before being aware of these requirements, which indicates that the obviousness of mother-to-daughter donation is not simply a matter of matching the requirements. While few of the interviewees explicitly elaborated on the reasons that mother-to-daughter donation is considered to be obvious, most of them did stress that it was not important, as such, to have their mother as a donor. Hence, the obviousness was not construed as a matter of maintaining bio-intimacy by keeping donation ‘in the family’, which Kroløkke and Nebeling61 suggest is enacted in UTx-IVF. Neither was it construed as a matter of providing a specific corporeal connectedness with a child-to-be and thus it, at least partly, contrasts with how women with a congenital absence of the uterus sometimes frame UTx-IVF.62

Instead, Emma’s and Melinda’s stories illustrate two other ways to understand the obviousness. In light of the ephemeral character of UTx-IVF, Emma’s story exemplifies how the obviousness can be construed as a matter of feasibility and proximity. It is possible that the knowledge that the organ is only temporary, and is not given to ensure survival but to enable gestational and genetic parenthood mitigates the concerns about anticipated contingencies with respect to embodiment, relationality and subjectivity that occur in other forms of organ donation.63–65 At the same time, by offering ‘reproductive potential’66 rather than survival, UTx-IVF may require other negotiations of expectations and relationships. As Melinda’s story exemplifies, the question of donation can make explicit expectations on the mother-daughter relationship, and it may call for consideration and negotiations, especially when the request is denied.

Similar to other reproductive transfers between related subjects67 and organ transfers from parents to their children,68 UTx-IVF involves many layers of relational entanglements that calls for management. However, the first pattern—I suggest—specifically brings into focus how the very possibility of transferring a uterus, make explicit certain specificities of UTx-IVF with respect to negations of and expectations on established entanglements between embodied subjects. It details how the ephemeral character of UTx-IVF may mitigate certain contingencies, and requires that uterus recipients already when considering such a transfer prepare to disentangle from the organ that they may receive. Furthermore, in light of the possibility of transferring a uterus, specific expectations on postmenopausal mothers, from others as well as from mothers themselves may emerge. In this way, the first pattern indicates how normative expectations on what motherhood involves may become enacted as UTx-IVF continues to unfold.

### Too much to ask for? Prospective donors and distributed entanglements

It said that you were supposed to get the uterus from a relative and then you started thinking: “Okay, if it’s me, who will give me one?” and then: “Oh my god, are you supposed to ask… or are they supposed to offer?” or … you know: “What if you are putting their life at risk, those who donate their uterus?”. You know there were lots of those questions, I think. Lots of them.

Ellen’s reflections above exemplify considerations of ‘whom’ and ‘how’ to ask for a donation that the interviewees emphasised as they told of their search for a donor. Central to these considerations was the idea that potential donors had to be ‘past the stage of having children’, which made the interviewees reluctant to ask sisters and friends who were about the same age as themselves. Mira, for example, explained that she had not told many friends about her condition since she was ashamed and she would, therefore, feel uncomfortable asking them. And it would be impossible to ask her sister:

I can’t ask my sister, because she hasn’t started a family yet. She has to have the chance to do that first.

They had not talked about this, Mira explained, but she had recently started to think about why her sister had not had children despite being in a stable relationship and having a permanent job. It feels as if she is waiting for me, Mira said. She continued:

I can’t ask her to volunteer. Because then…I’ll deprive her of something.

Mira’s reasoning showed how women of the same age as the interviewees were considered to be unapproachable as potential donors since donation would deprive them of the opportunity of pregnancy and a ‘normal’ life trajectory. In describing this ‘unapproachability’, the interviewees thus also drew on certain assumptions about the plans and desires of women of the same age when it comes to pregnancy and children. These assumptions, in turn, convey normative notions of how the life-course of young women is expected to be organised, making possible what Judith Halberstam calls a ‘reproductive temporality’69 with the building of a family at its core. This had to be taken into account when considering potential donors. Consequently, the interviewees emphasised, once your mother has been asked, you may not have a long list of other potential donors.

Related to how they emphasised that certain persons would be unapproachable, the interviewees emphasised, although in some cases implicitly, the reproductive purpose of the uterus. Victoria, for example, explained that to her, ‘that that’s the only thing that it (the uterus) is made for: children’. Mira reflected on the perspective of potential donors:

…it’s complicated to donate too, you can’t just…I think. But at the same time, I believe that many who could imagine themselves in this situation [of being in need of a uterus] would still think: “Well, you know, I don’t need it anymore, so why would I keep it? Then I might just as well donate it, if it’s possible”. That’s what I’d think, anyway. If there was a match with someone.

The interviewees’ emphasis on the importance of considering the reproductive temporality of potential donors—by underscoring that donors had to be ‘past the stage’ of having children—illustrates, I suggest, expectations on the life trajectory of women in reproductive age. The interviewees’ accounts express assumptions about a desire for pregnancy and (genetic and gestational) children, *and* that the uterus loses its meaning when a woman has passed reproductive age. When you are past the stage of having children, you are ‘done with’ your uterus, and thus it is possible that it is taken for granted that you are a potential donor. This way of reasoning also echoes ideas of function found in the medical discourse on uterus donation, where it is argued, for example, that—in contrast to other forms of live organ donation—the uterus has ‘exhausted (its) function for the donor, and its loss does not alter any present or future physiological function’.70

While uterus donation through such discourses becomes positioned as a rather simple matter of giving away an organ no longer in use, the interviewees indicated at the same time that it might not be such a simple matter after all. This became apparent as they described their difficulties in asking for a donation and the difficulties of potential donors in responding to such a request. Anna mentioned both the physical risks for the donor and relational contingencies that could emerge from the question of donation. She had not, so far, found a uterus that she would ‘feel good about having’ and she had not had anyone tell her “Hi, you can have mine”. She explained that she did try to raise the question with one family member but, Anna noticed, ‘she wasn’t really ready to go through with it’. Anna had also asked her mother.

And she [her mother] was OK with it, but then [a family member] started to influence me: “What if something happens?” and “Is it worth it?”, and then I felt guilty and…and then I told her: “No, I don’t want to go through with it”.

Donation, this account illustrates, may not be obvious to everyone and the risks may be considered to outweigh the possible benefits. Furthermore, in showing how the question of donation became a concern that involved not only Anna and her mother but also other family members, it illustrates how the question of donation can become a concern that involves others with whom the interviewee is intimately entangled. The management of entanglements that the question of donation instigated was thus not confined to the recipient-donor relationship, but distributed across the intimate entanglements of which they were part.

Like Anna, other interviewees emphasised that a uterus is not something that you are offered on a daily basis. Sara, for example, said that it is not ‘like you go around asking “Could you consider being my donor?”’ However, two close relatives had indeed offered to donate as soon as they found out that Sara was looking for a uterus. They both said, ‘Take mine!’, Sara recalled, but pointed out that: ‘That’s an easy thing to say’, and that one of them was ‘terrified’ of transplantation. As Sara’s mother had offered to donate at an early stage, Sara had never followed up on her relatives’ offer. Like Anna, Sara expressed concern about risks, although in her case she emphasised the relational risks more than the physical ones.

But… because I don’t want her [her mother] to feel… guilty. And I don’t think she will, *because she is so fascinated by the whole thing*. What she is afraid of is that she will not be…approved. So that she’ll disappoint me. But she could never disappoint me! But I don’t think that she sees that, herself. And I can understand that, in a way.

While Sara elsewhere made it quite clear that her mother had always expressed a willingness to donate, she indicates in this account that the offer was accompanied by certain relational riders, such as the risk of guilt and disappointment. Hence, the main issue was not what transplantation might do to the recipient’s or the donor’s body, but what a failed transplantation would do to the already established relational mother-daughter entanglement.

Although the first pattern comprises accounts that positioned it as obvious and fairly uncomplicated to disentangle the uterus from the donor’s embodied self, the examples of the second pattern, I suggest, illustrate that physical risks for the donor as well as emotional risks—such as troublesome feelings of guilt and responsibility for the organ after transplantation—were taken into account when considering potential donors. While Anna’s account shows how these concerns were distributed and negotiated within the family, Sara’s account reveals a worry that feelings of guilt and disappointment would disrupt her relationship with her mother, and thus impede relational re-entanglement post-transplantation.

The reproductive function of the uterus was often emphasised as the interviewees considered whom they felt it was possible to approach with the question of donation. Yet, as the interviewees described their deliberations, it became clear that not everyone for whom having children is no longer relevant is approachable. It might prove not to be easy to disentangle from the uterus, and some might find donation truly terrifying. To ask someone close to you for a uterus—be it your mother or someone else—requires considerations not only of the physical risks but also of the effect of donation on the already close relational entanglement between the one asking and the one being asked. As the very question of donation and the considerations that come with it may involve others with whom the one asking and the one being asked are entangled, the question of donation requires efforts to mitigate anticipated disruptions of established entanglements. Hence, it was—again—entanglements that preceded, rather than resulted from, donation that required management.71

### Resolutions? Unexpected entanglements and disentanglements

I was told that “Yes, you could very well be a candidate [for UTx-IVF], but you do need to find… to have a donor”. So the clock was ticking for me. Really. It was very stressful. We had kind of explored all possibilities.

Like Bella, in the account above, several of the interviewees expressed that a failure to find a donor had been, or would be, stressful. Some of those who had been actively looking for a donor had continued to search after failing to find one among their closest relatives. They frequently mentioned that they had hoped that there was a register for potential donors, or at least a way in which women prepared to donate could express their interest.

Since UTx-IVF still only is performed as part of research in Sweden, structures available for other kinds of organ donation, such as donor registries, have not been established. At the same time, the fact that UTx-IVF was only performed as research might have allowed for flexibility with respect to the requirements. Bella, for example, who even had looked into the possibility of placing an advertisement in the newspaper in order to find a donor, was eventually approached by an acquaintance who volunteered to investigate the possibility of becoming her donor. Also Mira, who had been quite confident that one of her family members could become a donor, later found out that none of them would be accepted as donors for medical reasons. When Mira had been informed of this, she mentioned it to an older friend who immediately said that she was willing to investigate if she could become Mira’s donor. Mira recalled her friend emphasising:

“And if it turns out that we match then, then I would like to donate”.

L: Oh, what did you think about that?

Mira: *Eee… what did I think…*

L: Or felt?

Mira: It, it… I think it was more like… “Really, do you know what you are getting yourself into?” Eee… And this… it is not… it is really not… it is really not just… it not a tiny thing, you know. Its… you’re not just giving some blood…but it is… you’re really taking something out from your body. But she’s been… all this time she’s been very aware of what it is that she has done. And, you know, have been reading up on everything. […] That’s what she said. “So I want to do this”. And that’s been quite nice for me, because I’ve never asked her. While mom…with her it’s been more…taken for granted that she would donate. […] Well she’s…she’s *a fairy godmother really*. Because if she hadn’t volunteered, I don’t know where I would have found someone.

Bella’s and Mira’s stories demonstrate how access to transplantation, when it was required that one brings one’s own donor, became to some extent a matter of chance, but it still depended also on established relationships in which one felt comfortable enough to share one’s story. Both of them had happened to share their story with the right person at the right time, and their search for a donor thus resulted in unexpected entanglements involving a donor who was not a relative and for whom donation was not taken for granted. Mira’s story further demonstrated that in some cases it might be preferable to have another donor than your own mother. While there may very well be strong medical reasons for having your mother as a donor, on a relational level it may feel easier to be offered a uterus from someone who is not ‘taken for granted’ as a donor. Then, Mira indicated, you can feel more certain that it is something that the donor has considered carefully. By this, I do not suggest that a mother’s decision to donate a uterus to her daughter is never carefully thought through. Mira’s example, however, shows that being offered a uterus by someone not so close demonstrates that the obviousness of mother-to-daughter donation is not always easy. Hence, being offered a uterus from someone not as close may reduce the suspicion that the decision to donate had been a decision without reflection, rather than one that was well thought through.

For some of the interviewees, the quest for a uterus came to an end for one of several possible reasons: it was not possible to enter the research project at the time, they decided to pursue another route to parenthood, or because they failed to find a donor. Emma, who—as part of the first pattern—emphasised that she had always assumed that her mother would be able to donate, eventually discovered that her mother would not be accepted as a donor because of her previous medical history.

Emma: So we had to send them [the medical records] to them, just to check, basically. And we said that we do understand what this means, we can read it ourselves. But they confirmed it quite quickly. That it wouldn’t work. If you don’t have someone else who could consider donating. But…we haven’t, at the moment. So it ended pretty quickly too. At the same time as it was a bit drawn out.

To find out that she could not have a donation from her mother was ‘as tough as hell’, Emma said, especially because her partner and she felt ‘that our premises were really good’. They thought, Emma said, that ‘this will work out fine, for sure, because we’re young and fertile and my mom is in good shape’.

So it felt like: “We’ll fix this!”, perhaps. Or yes. But then I got really sad, when we found out. Or I’m still really sad, but now it’s like another phase, in a way. Back then, it was more the initial shock.

Emma’s account describes the difficulty she had when she found out that the person she had taken for granted to become a donor would not be accepted. The realisation that transplantation was not an option, at least not at that time, took Emma back to square one and the feelings she had had as a teenager when she found out about her condition. She started therapy to try to deal with her experiences and once again figure out how to live without a uterus. While Emma did not describe the result of the failure to access transplantation as a disentanglement of her and her mother’s relationship, it did indeed disrupt an expected life trajectory and thus had an impact on her everyday life. The sorrow that she described suggests that support is needed to undergo the process of transplantation, and to manage the disruption to one’s expected trajectory that can occur when one is not accepted for transplantation.

The resolutions that the third pattern highlighted did not necessarily involve transplantation and the birth of a child. Instead, the third pattern involved descriptions of different resolutions to the interviewees’ quest for a uterus donor. Bella’s and Mira’s stories told that when they had exhausted all alternatives to find a donor among closest family and friends, the search became a matter of whom they felt they could, or happened to, share their story with. However, it should be kept in mind that several interviewees were, for different reasons, still seeking resolution. Some of them had decided to actively pursue other routes to parenthood; others were still considering whether to continue to pursue UTx-IVF. Previous research72–74 has described the efforts required to make sense of subjectivity, embodiment, and relations when ARTs (such as IVF) fail. For the interviewees in the present study, the realisation that UTx-IVF was not an alternative required, as Emma’s story demonstrated, negotiations with one’s disrupted life trajectory.

The third pattern, thus, illustrated support in different ways. On the one hand, in demonstrating how finding a donor might become a matter of chance, Bella’s and Mira’s accounts showed how support could appear unexpectedly when sharing one’s story. It also showed how this support, in their cases, ultimately resolved their search for a donor. On the other hand, Emma’s account detailed the need for support not to find a donor but to deal with the realisation that there was no donor to be found. Hence, Emma’s account underscored the need for healthcare to offer support and care also for those who are not accepted for transplantation, who have not found resolution and when the lack of resolution brings struggles and pain from the time of diagnosis to life.

## Conclusions

In many respects, my analysis of the interviewees’ quest for a donor echo findings from previous research on the relational complexities and contingencies of live, related, organ donation. It shows that there is a risk of pressure and emotional burden on the donor and recipient alike,75–77 detail the special circumstances of live organ donation between family members,78 and clarify the difficulties associated with asking someone close to you for an organ.79 However, it also more specifically identifies a discourse that signals an obviousness of mother-to-daughter donation. To conclude, I address norms that are enacted through the emphasis of this obviousness of mother-to-daughter donation and the value of staying sensitive to the management of entanglements that unfold when asking someone for a donation.

When discussing the obviousness of mother-to-daughter donation as it is expressed in the interviewees’ accounts, some particular aspects of UTx-IVF and the Swedish context must be kept in mind. As noted above, in the Swedish context UTx-IVF is only conducted as research. There is, at the moment, no donor registry, and a woman who wants to undergo UTx-IVF has had to bring a donor for whom the period of having children is over and to whom she is related.80 Finally, and in contrast to other live organ donations, only individuals with a uterus can volunteer as donors in UTx-IVF, meaning that it is (primarily) women who can become donors.81 Against this backdrop, the obviousness of first asking your mother might not seem surprising. Likewise, in the light of previous studies in which parents describe kidney donation to their child as ‘natural’ and are brought to do so by witnessing their child’s suffering,82 it should not be surprising that the interviewees described it to be obvious for their mothers to volunteer as a donor.

However, when considering the ‘obviousness’ of mother-to-daughter donation in more detail, it also becomes clear how it expresses expectations of what a mother ‘should’ be willing to do for her child and the relational conundrums that can arise when such expectations are not met. In the above analysis, this was brought out through an emphasis of the reproductive functionality of the uterus which positioned the uterus a container for carrying a child. That is, when the period of having children is over for a woman, it might be considered that she no longer needs or wants a uterus. The quest for a uterus can thus be seen to rely on norms about motherhood that signal expectations about responsibilities in the relational entanglement between mother and daughter, and on norms about female embodiment which signal that a person who has passed reproductive age no longer needs a uterus. The analytic patterns specifically revealed how these norms shape considerations of whom to ask for a donation, what response to expect when doing so, and contribute to position mother-to-daughter donation as ‘obvious’.

By this, I do not mean to dismiss that many women might indeed feel that they do not need their uterus anymore and that it thus may be obvious for them to donate. Women might also want to volunteer for non-directed uterus donation if given the opportunity. A survey of Swedish women’s attitudes to ARTs, for example, demonstrated a preference for UTx-IVF over surrogacy83 and a study of attitudes towards UTx-IVF in the USA suggested that there is a strong interest in donating a uterus, even for non-directed donors.84 It has also been argued85 that UTx-IVF poses ‘less of an ethical dilemma than liver or kidney donation’ precisely because of the ‘exhausted function’ of the uterus after reproductive age. Nevertheless, we need to take seriously that knowledge is scarce about how women reason about uterus donation. If seeking to understand how women reason about ART in general and UTx-IVF in particular, I hold that further knowledge is needed to avoid assumptions about women’s feelings towards their uterus and their preferences with respect to donating it.

The ‘obviousness’ of mother-to-daughter donation identified above can also be seen to obscure the many meanings and emotions connected to the uterus, expressed for example by those who are born without one, and who thus seek UTx-IVF86or by hysterectomised women who have had their uterus removed.87 Such experiences point to the importance of not considering the uterus merely as a container meant to carry a pregnancy, but to further investigate the emotional significance of this particular body part in relation to different cultural understandings of and expectations on gendered bodies. In particular, the above analysis above has showed—in correspondence with problematisations of UTx-IVF as inscribing moral imperatives and gender norms88—how important it is to take into consideration what norms, when enacted in medical practice, *do* when discussing the ethics of UTx-IVF. By this, I do not mean that issues discussed in the ethical debate over such topics as risk-benefit, priority-setting and reproductive liberty should be set aside. Instead, I suggest that we can develop them further by taking into account participants’ experiences of risks and benefits and how they intertwine with societal and gendered norms and expectations, enacted for example through discourses that emphasise the reproductive functionality of the uterus. In this manner, ethical discussions can become more sensitive to how norms of motherhood and reproductive functionality—when expressed in ways that position uterus donation as obvious and the uterus as a body part that can be easily spared—shape how UTx-IVF is approached.89 This is not a matter of simply detecting undue pressure on potential donors, but of analysing how the normative underpinnings of UTx-IVF create contexts in which certain choices become accepted or given priority over others. It is a way of considering how opportunities to resist or challenge such norms in different contexts become limited, while at the same time seeking to avoid portraying women as duped by normative understandings of motherhood and reproduction.90 It means paying close attention to the situatedness of UTx-IVF and those subjects—patients, family members, healthcare staff—who engage in this development. That is, while all of my interviewees were Swedish citizens with access to Swedish public healthcare, the specificities of their embodied histories, relations and situations still shaped their approaches and opportunities to access to UTx-IVF. Their accounts of UTx-IVF by no means represent those of every woman who finds herself ‘in need’ of a uterus. The meanings accorded to and the access granted to this kind of medical innovation would most certainly, differ greatly if analysing stories from queer or non-heterosexual individuals, individuals located in other healthcare systems or let alone individuals in the global south. To take into consideration norms enacted in UTx-IVF requires therefore that we understand accounts of UTx-IVF as shaped and told in particular embodied location.

Finally, my intention in this article has not been to argue for or against the development of UTx-IVF, but to—in line with Scheper-Hughes91—contribute to making visible the less visible contingencies that risk being overlooked when, for example, the discussion of risk focuses on the medical consequences and complications of UTx-IVF. While mother-to-daughter donation was indeed construed as obvious by the interviewees, their quest for a donor was not necessarily straightforward. To ask someone—be it a mother, sister or someone else—for a uterus involves several considerations and negotiations. As the interviewees described their quest, they showed that relational conundrums not only may follow but may precede transplantation, and may linger when transplantation does not take place or is not successful. Undesirable relational disentanglements that unfolded from the question of donation created an anticipation of feelings of guilt or disappointment. While undesirable disentanglement was not perceived as an imminent threat, it was still construed as a serious concern that must be managed, together with any physical consequences for the donor. In the light of this, some interviewees argued, the offer of a uterus from someone with whom you are not already closely relationally entangled might bring fewer complications. As such an offer was not taken for granted, it seemed to pose less of a threat to already established relationships. In this way, the analysis demonstrated the difficulties faced when asking for a donation and thus made it clear how access to a donor is a matter of what networks are available and how open one is about the condition.

In the Swedish context, it remains to be decided whether this requirement will be maintained if (or when) the procedure comes to be offered as part of the general healthcare system, not solely in a research context. Likewise, international discussions of live versus deceased donation in UTx-IVF continue and the specific regulation of UTx-IVF, for example, with respect to the provision of donors, remains to be decided in several countries. Until such issues are resolved, the contingencies raised in the analysis above show that it is a matter of urgency to examine what the requirement of bringing your own donor entails in terms of creating stress for potential recipients, and how it contributes to disparities in access to transplantation. I suggest that by taking seriously the occurrence and anticipation of such relational conundrums and the management of entanglements that may proceed and follow donation the ethical debate on UTx-IVF can become more attuned to the experiences of those whom UTx-IVF is intended to help. In doing so, it can reach beyond discussions of (medical) complications associated with the transplantation process92 and expand our understanding of what is at stake as UTx-IVF advances.

## Data Availability

No data are available. The data analysed in this article consists of deidentified interview data. It is not available to anyone outside the research group.

## Notes

1. In medical literature, it is sometimes approximated that AUFI affects 1/500 women of fertile age, which is said to correspond to 200 000 women in Europe. Niclas Kvarnström et al. (2017), ‘Live Donors of the Initial Observational Study of Uterus Transplantation—Psychological and Medical Follow-up Until 1 Year After Surgery in the 9 Cases’, *Transplantation* 101, no. 3 (1 March 2017): 664–70, 10.1097/TP.0000000000001567. However, it has also been noted that knowledge gaps exist with respect to the incidence of uterine anomalies, see Grigoris F Grimbizis et al. (2001), ‘Clinical Implications of Uterine Malformations and Hysteroscopic Treatment Results’, *Human Reproduction* Update 7, no. 2 (1 March 2001): 161–74, 10.1093/humupd/7.2.161.

2. The first transplantations in the Swedish UTx-IVF research project were conducted in 2013, when nine Swedish women received a uterus from a live donor who was either related to or a friend of the recipient, Mats Brännström, (2019), ‘Chapter 23 - Uterine Transplantation’, in Human Reproductive and Prenatal Genetics, ed. Peter C. K. Leung and Jie Qiao (Academic Press), 515–25, 10.1016/B978-0-12-813570-9.00023-1. To take part in the Swedish live donor UTx-IVF research project, the donor, the recipient, and the recipient’s partner undergo an extensive assessment (including both psychological tests and physical matching) and, if the participants are accepted to proceed, eggs are harvested from the recipient and subsequently fertilized in vitro using her partner’s sperm. When a satisfactory number of high-quality embryos have been produced and frozen, the cervix, the uterus and two major blood vessels are transplanted from the donor and connected to the vagina, but not to the ovaries, of the recipient. To avoid rejection episodes, the recipient is given immunosuppressive treatment and about a year later, if she has started menstruating and the organ is stable, embryo transfers are initiated. In the event of pregnancy, the child is delivered by cesarean section. If she wishes to and if assessed to be medically fit, the recipient may attempt to become pregnant a second time. However, it is not intended that the transplanted uterus last for the lifetime of the recipient. After a maximum of two full-term pregnancies, the uterus will be removed in order to minimize the time spent on immunosuppressing treatment. See, Mats Brännström et al. (2015) ‘Livebirth after Uterus Transplantation’, *Lancet* 385, no. 9968 (14 February 2015): 607–16, 10.1016/S0140-6736(14)61728-1. Finally, it should be noted that in Sweden UTx-IVF is currently funded solely as a research procedure and is not accessible through the general health care system.

3. Mats Brännström et al. (2019), ‘Global Results of Human Uterus Transplantation and Strategies for Pre-Transplantation Screening of Donors’. *Fertility and Sterility* 112, no. 1 (2019): 3–10. 10.1016/j.fertnstert.2019.05.030.

4. C Petrini et al. (2017), ‘Ethical Issues in Uterine Transplantation: Psychological Implications and Informed Consent’, *Transplantation Proceedings* 49, no. 4 (1 May 2017): 707–10, 10.1016/j.transproceed.2017.02.013; Brännström et al., ‘Livebirth after’

5. This is the case when a woman donates her uterus to her genetic and gestational daughter.

6. Transplantations using deceased donors have also recently been initiated. See Sara Frisk (2019), ‘Nu planeras första livmodern från en avliden donator att transplanteras i Sverige’, SVT Nyheter, 9 January 2019, Vetenskap, https://www.svt.se/nyheter/vetenskap/under-varen-transplanteras-forsta-livmodern-fran-avliden-donator-i-sverige.

7. Sahlgrenska Academy, Gothenburg University, ‘Prerequisites for Transplantation’, Accessed December 14 2018, https://sahlgrenska.gu.se/english/research/uterus/process.

8. Sahlgrenska Academy, Gothenburg University (2018), ‘Criteria for Donors’, Accessed December 14 2018, https://sahlgrenska.gu.se/english/research/uterus/process/donor

9. Bruno Bethany and Kavita Shah Arora (2018), ‘Uterus Transplantation: The Ethics of Using Deceased Versus Living Donors’, *The American Journal of Bioethics* 18, no. 7 (3 July 2018): 6–15, 10.1080/15265161.2018.1478018; Vincent Lavoué et al. (2017), ‘Which Donor for Uterus Transplants: Brain-Dead Donor or Living Donor? A Systematic Review’, *Transplantation* 101, no. 2 (1 February 2017): 267–73, 10.1097/TP.0000000000001481; Heidi Mertes and Kristof Van Assche (2018), ‘UTx With Deceased Donors Also Places Risks and Burdens on Third Parties’, *The American Journal of Bioethics* 18, no. 7 (3 July 2018): 22–24, 10.1080/15265161.2018.1478029.

10. BP Jones et al. (2019), ‘Uterine Transplantation in Transgender Women’, *BJOG: An International Journal of Obstetrics & Gynaecology* 126, no. 2: 152–56, 10.1111/1471-0528.15438; Lars Sandman (2018), ‘The Importance of Being Pregnant: On the Healthcare Need for Uterus Transplantation’, *Bioethics* 32, no. 8 (1 October 2018): 519–26, 10.1111/bioe.12525; Stephen Wilkinson and Nicola Williams (2016), ‘Should Uterus Transplants Be Publicly Funded?’, *Journal of Medical Ethics* 42, no. 9: 559–65, 10.1136/medethics-2015-102999.

11. Amel Alghrani (2018a), Regulating Assisted Reproductive Technologies: New Horizons (Cambridge, United Kingdom; New York, NY: Cambridge University Press, 2018); Amel Alghrani (2018b), ‘Uterus Transplantation in and beyond Cisgender Women: Revisiting Procreative Liberty in Light of Emerging Reproductive Technologies’, *Journal of Law and the Biosciences* 5, no. 2 (10 July 2018): 301–28, https://doi.org/Online only. DOI: 10.1093/jlb/lsy012; Cristie Cole Horsburgh (2017), ‘A Call for Empirical Research on Uterine Transplantation and Reproductive Autonomy’, *Hastings Center* Report 47, no. S3: S46–49, 10.1002/hast.795.

12. Megan Allyse (2018), ‘“Whole Again”: Why Are Penile Transplants Less Controversial Than Uterine?’, *The American Journal of Bioethics* 18, no. 7 (3 July 2018): 34–35, 10.1080/15265161.2018.1478044; Ruby Catsanos, Wendy Rogers, and Mianna Lotz (2013), ‘The Ethics of Uterus Transplantation’, *Bioethics* 27, no. 2: 65–73, 10.1111/j.1467-8519.2011.01897.x; Lisa Guntram and Kristin Zeiler (2019), ’The Ethics of the Societal Entrenchment-approach and the Case of Live Uterus Transplantation-IVF’, *Medicine, Health Care and Philosophy* 22, no. 4: 557–71. 10.1007/s11019-019-09891-w; Mianna Lotz (2018), ‘Uterus Transplantation as Radical Reproduction: Taking the Adoption Alternative More Seriously’, *Bioethics* 32, no. 8: 499–508, 10.1111/bioe.12490; Mabel, Farrell, and Tzakis, ‘On Gender and Reproductive’.

13. See for example, Ruth M Farrell, Rebecca Flyckt, and Tommaso Falcone (2018), ‘The Call for a Closer Examination of the Ethical Issues Associated with Uterine Transplantation’, *Journal of Minimally Invasive Gynecology* 25, no. 6 (1 September 2018): 933–35, 10.1016/j.jmig.2018.07.016; Hilary Mabel, Ruth M Farrell, and Andreas G Tzakis (2018), ‘On Gender and Reproductive Decision-Making in Uterine Transplantation’, *The American Journal of Bioethics* 18, no. 7 (3 July 2018): 3–5, 10.1080/15265161.2018.1489655

14. Nicola Jane Williams, Rosamund Scott, and Stephen Wilkinson (2018), ‘The Ethics of Uterus Transplantation’, *Bioethics* 32, no. 8 (1 October 2018): 478–80, 10.1111/bioe.12530.

15. Giuliano Testa and Liza Johannesson (2017), ‘The Ethical Challenges of Uterus Transplantation’, *Current Opinion in Organ Transplantation* 22, no. 6 (December 2017): 593–97, 10.1097/MOT.0000000000000467.

16. Petrini et al., ‘Ethical Issues’.

17. Michael Olausson et al. (2014), ‘Ethics of Uterus Transplantation with Live Donors’, *Fertility and Sterility* 102, no. 1 (1 July 2014): 40–43, 10.1016/j.fertnstert.2014.03.048.

18. Iori Kisu et al. (2013), ‘Risks for Donors in Uterus Transplantation’, *Reproductive Sciences* 20, no. 12 (1 December 2013): 1406–15, 10.1177/1933719113493517; Olausson et al., ‘Ethics’.

19. Kisu et al., ‘Risks for Donors’.

20. See however Jean Elson (2002), ‘Menarche, Menstruation, and Gender Identity: Retrospective Accounts from Women Who Have Undergone Premenopausal Hysterectomy’, *Sex Roles* 46, no. 1 (1 January 2002): 37–48; Kari Nyheim Solbrække and Hilde Bondevik (2015), ‘Absent Organs—Present Selves: Exploring Embodiment and Gender Identity in Young Norwegian Women’s Accounts of Hysterectomy’, *International Journal of Qualitative Studies on Health and Well-Being* 10, no. 1 (1 January 2015): 26720, 10.3402/qhw.v10.26720.

21. With respect to the intended child, benefits discussed have concerned the benefit of being born at all Lisa Guntram and Nicola Williams (2018), ’Positioning uterus transplantationas a ’more ethical’ alternative to surrogacy: Exploring symmetries between uterustransplantation and surrogacy through analysis of a Swedish government white paper’, *Bioethics* 32, no. 8: 509–18. 10.1111/bioe.12469, while discussions of risks have predominantly addressed rejection episodes of the uterus during pregnancy, effects of immunosuppression, pre-term delivery and preeclampsia see for example Kavita Shah Arora and Valarie Blake (2015), ‘Uterus Transplantation: The Ethics of Moving the Womb’, *Obstetrics & Gynecology* 125, no. 4 (April 2015): 971–74, 10.1097/AOG.0000000000000707; Olausson et al., ‘Ethics’; Testa and Johannesson, ‘The Ethical’.

22. Allyse, ‘Whole Again’; Catsanos et al, ‘The Ethics’; Lotz, ‘Uterus’; Mabel, Farrell, and Tzakis, ‘On Gender and Reproductive’; [self-reference removed during review].

23. Bruno and Arora, ‘Uterus Transplantation’; Lavoué et al., ‘Which Donor’; Mertes and Van Assche, ‘UTx With Deceased’.

24. Susan McKinnon (2015), ‘Productive Paradoxes of the Assisted Reproductive Technologies in the Context of the New Kinship Studies’, *Journal of Family Issues* 36, no. 4 (1 March 2015): 461–79, 10.1177/0192513X14563799; Katherine Carroll and Charlotte Kroløkke (2018), ‘Freezing for Love: Enacting “Responsible” Reproductive Citizenship through Egg Freezing’, *Culture, Health & Sexuality* 20, no. 9 (2 September 2018): 992–1005, 10.1080/13691058.2017.1404643; Sarah Franklin (1997), Embodied Progress: A Cultural Account of Assisted Conception (London: Routledge); Gemma Orobitg and Carles Salazar (2005), ‘The Gift of Motherhood: Egg Donation in a Barcelona Infertility Clinic’, *Ethnos* 70, no. 1 (1 March 2005): 31–52, 10.1080/00141840500048532; Nancy Scheper-Hughes (2000), ‘The Global Traffic in Human Organs’, *Current Anthropology* 41, no. 2: 191–224, 10.1086/300123.

25. Bob Simpson (2013), ‘Managing Potential in Assisted Reproductive Technologies: Reflections on Gifts, Kinship, and the Process of Vernacularization’, *Current Anthropology* 54, no. S7 (1 October 2013): 88, 10.1086/670173.

26. McKinnon, ‘Productive Paradoxes’, 466.

27. Robert A Crouch and Carl Elliott (1999), ‘Moral Agency and the Family: The Case of Living Related Organ Transplantation’, *Cambridge Quarterly of Healthcare Ethics* 8, no. 03: 275–87.

28. Nancy Scheper-Hughes (2007), ‘The Tyranny of the Gift: Sacrificial Violence in Living Donor Transplants’, *American Journal of Transplantation* 7, no. 3 (1 March 2007): 507–11, 10.1111/j.1600-6143.2006.01679.x.

29. Deborah Ummel and Marie Achille (2016), ‘Transplant Trajectory and Relational Experience Within Living Kidney Dyads’, *Qualitative Health Research* 26, no. 2 (1 January 2016): 194–203, 10.1177/1049732315570128.

30. Paul Gill et al. (2008), ‘Gift Exchange and Organ Donation: Donor and Recipient Experiences of Live Related Kidney Transplantation’, *International Journal of Nursing Studies* 45, no. 11 (November 2008): 1607–17; Ingrid B de Groot et al. (2012), ‘Decision Making around Living and Deceased Donor Kidney Transplantation: A Qualitative Study Exploring the Importance of Expected Relationship Changes’, *BMC Nephrology* 13 (7 September 2012); Christina Papachristou et al. (2009), ‘Living Donor Liver Transplantation and Its Effect on the Donor-Recipient Relationship – a Qualitative Interview Study with Donors’, *Clinical Transplantation* 23, no. 3 (July 2009): 382–91; Karen Schipper et al. (2014), ‘Sweet and Sour after Renal Transplantation: A Qualitative Study about the Positive and Negative Consequences of Renal Transplantation’, *British Journal of Health Psychology* 19, no. 3 (September 2014): 580–91, 10.1111/bjhp.12057; Johanna Spiers, Jonathan A Smith, and Martin Drage (2016), ‘A Longitudinal Interpretative Phenomenological Analysis of the Process of Kidney Recipients’ Resolution of Complex Ambiguities within Relationships with Their Living Donors’, *Journal of Health Psychology* 21, no. 11 (1 November 2016): 2600–2611, 10.1177/1359105315581070.

31. Alison K. Crombie and Patricia M. Franklin (2006), ‘Family Issues Implicit in Living Donation’, *Mortality, Current issues in organ donation and transplantation*, 11, no. 2 (May 2006): 196–210, 10.1080/13576270600616011; H M Kärrfelt et al. (2000), ‘Renal Transplantation in Children: Psychological and Donation-Related Aspects from the Parental Perspective’, *Pediatric Transplantation*; Annette Lennerling et al. (2004), ‘Motives for Becoming a Living Kidney Donor’, *Nephrology Dialysis Transplantation* 19, no. 6 (6 January 2004): 1600–1605, 10.1093/ndt/gfh138; Kristin Zeiler, Lisa Guntram, and Anette Lennerling (2010), Moral tales of parental living kidney donation: a parenthood moral imperative and its relevance for decision making’, *Medicine, Health Care and Philosophy* 13, no. 3: 225–36. 10.1007/ s11019-010-9238-3.

32. Margareta A Sanner et al. (2011), ‘The Kidney Recipient’s Path to Transplantation: A Comparison between Living and Deceased Kidney Donor Recipients in Stockholm, Sweden’, *Nephrology Dialysis Transplantation* 26, no. 3 (1 March 2011): 1053–57, 10.1093/ndt/gfq462.

33. See, for example, Schipper et al., ‘Sweet and Sour; Spiers, Smith, and Drage, ‘A Longitudinal Interpretative’.

34. de Groot et al. ‘Decision Making’.

35. Michel Callon (1998), ‘Introduction: The Embeddedness of Economic Markets in Economics’, *The Sociological Review* 46, no. 1_suppl (1 May 1998): 1–57, 10.1111/j.1467-954X.1998.tb03468.x.

36. Sarah N Boers et al. (2019), ‘Organoids as Hybrids: Ethical Implications for the Exchange of Human Tissues’, *Journal of Medical Ethics* 45, no. 2 (1 February 2019): 131–39, 10.1136/medethics-2018-104846.

37. Catherine Waldby and Robert Mitchell (2006), Tissue Economies: Blood, Organs, and Cell Lines in Late Capitalism (Duke University Press), 68.

38. Waldby and Mitchell, ‘Tissue Economies’ 68.

39. Callon, ‘Introduction’, 36.

40. Callon, ‘Introduction’, 37.

41. Callon, ‘Introduction’, 18.

42. See also; Boers et al. ‘Organoids as Hybrids’; Klaus Hoeyer (2013), ‘What Is a Human Body?’, In Exchanging Human Bodily Material: Rethinking Bodies and Markets (Springer, Dordrecht), 65–98, 10.1007/978-94-007-5264-1_3; Kristin Zeiler (2019), ‘Bodily Sharing’, In Sharing Our Bodies – Phenomenology, Bioethics, and Critical Medical Humanities, forthcoming.

43. Zeiler, ‘Bodily Sharing’.

44. Scheper-Hughes, ‘The Tyranny’.

45. Margrit Shildrick et al. (2009), ‘Troubling Dimensions of Heart Transplantation’, *Medical Humanities* 35, no. 1 (6 January 2009): 35–38, 10.1136/jmh.2008.001073.

46. Cf. Zeiler, ‘Bodily Sharing’.

47. See Ministry of Health and Social Affairs (2006), Lag (2006: 351) Om Genetisk Integritet m.m., vol. 351 (Swedish Code of Statutes).

48. See Socialstyrelsen (2014), ‘Adoption. Handbok För Socialtjänsten’ (Stockholm: The National Board of Health and Welfare), 16, https://www.socialstyrelsen.se/publikationer2014/2014-8-1.

49. See Laura L Ellingson (2006), ‘Embodied Knowledge: Writing Researchers’ Bodies Into Qualitative Health Research’, *Qualitative Health Research* 16, no. 2 (February 2006): 298–310, 10.1177/1049732305281944 and Maree Burns (2003), ‘I. Interviewing: Embodied Communication’, *Feminism & Psychology* 13, no. 2 (1 May 2003): 229–36, 10.1177/0959353503013002006.

50. Lisa Guntram (2013). ’"Differently Normal" and "Normally different": Negotiations of Female Embodiment in Women’s Accounts of ’Atypical’ Sex Development’, *Social Science & Medicine* 98: 232–8. 10.1016/j.socscimed.2013.09.018. Lisa Guntram, (2013), ’Creating, Maintaining and Questioning (Hetero)relational Normality in Narratives about Vaginal Reconstruction’, *Feminist Theory* 14, no. 1: 105–21. 10.1177/146470011246857; Lisa Guntram (2018), ’Hooked on a Feeling? Exploring Desires and ’Solutions’ in Infertility Accounts Given by Women with ’Atypical’ Sex Development’, *Health* 22, no. 3: 259– 76. 10.1177/1363459317693403.

51. See, for example, Cindy M Bird (2005), ‘How I Stopped Dreading and Learned to Love Transcription’, *Qualitative Inquiry* 11, no. 2 (4 January 2005): 226–48, 10.1177/1077800404273413; Christina Davidson (2009), ‘Transcription: Imperatives for Qualitative Research’, *International Journal of Qualitative Methods* 8, no. 2 (1 June 2009): 35–52, 10.1177/160940690900800206; Blake D Poland (1995), ‘Transcription Quality as an Aspect of Rigor in Qualitative Research’, *Qualitative Inquiry* 1, no. 3 (9 January 1995): 290–310, 10.1177/107780049500100302.

52. Virgina Braun and Victoria Clarke (2006), ‘Using Thematic Analysis in Psychology’, *Qualitative Research in Psychology* 3, no. 2 (1 January 2006): 77–101, 10.1191/1478088706qp063oa.

53. See also Corinne Squire (2008), ‘Experience-Centred and Culturally – Oriented Approaches to Narrative’, In Doing Narrative Research, ed. Molly Andrews, Corinne Squire, and Maria Tamboukou (Thousand Oaks CA: Sage), 42.

54. Braun and Clarke, ‘Using Thematic’.

55. Lisa Guntram and Ericka Johnson (2018), ’Feminist Approaches to Using Other People’s Words: Two Examples’, SAGE Research Methods Cases Part 2, 1–12: Sage. 10.4135/9781526443236.

56. See, for example, Guntram, ’"Differently Normal"’; Guntram, ’Creating, Maintaining’; Guntram (2018), ’Hooked on’.

57. Hard brackets in quotes indicate that a section or word has been removed to ensure confidentiality or to increase readability (eg, as in the case of studders or sidetracks) or provide explanatory information.

58. Crombie and Franklin, ‘Family Issues’; Karrfelt, Berg, and Lindblad, ‘Renal Transplantation’; Lennerling et al., ‘Motives for Becoming’; Zeiler, Guntram and Lennering, ’Moral Tales’.

59. *xx* indicates laughter.

60. Petrini et al., ‘Ethical Issues’.

61. Charlotte Kroløkke and Michael Nebeling Petersen (2017), ‘Keeping It in the Family: Debating the Bio-Intimacy of Uterine Transplants and Commercial Surrogacy’, In Bioethics Beyond Altruism Donating and Transforming Human Biological Materials, ed. Rhonda M. Shaw (Palgrave Macmillan), 10.1007/978-3-319-55532-4.

62. Guntram (2018), ’Hooked on’.

63. Schipper et al., ‘Sweet and Sour’.

64. Spiers, Smith, and Drage, ‘A Longitudinal Interpretative’.

65. Papachristou et al., ‘Living Donor Liver’.

66. Simpson, ‘Managing Potential’.

67. C.f. Zeiler, ‘Bodily Sharing’.

68. Crombie and Franklin, ‘Family Issues’; Karrfelt, Berg, and Lindblad, ‘Renal Transplantation’; Lennerling et al., ‘Motives for Becoming a Living Kidney Donor’; Zeiler, [self-reference removed during review].

69. Judith Halberstam (2005), In a Queer Time and Place: Transgender Bodies, Subcultural Lives, 5, http://www.loc.gov/catdir/toc/ecip0422/2004018151.html.

70. Testa and Johannesson, ‘The Ethical’, 595.

71. Callon, ‘Introduction’.

72. Throsby, (2004), ‘Negotiating Normality When IVF Fails’, In Considering Counter Narratives: Narrating, Resisting, Making Sense, ed. Michael Bamberg and Molly. Andrews (Amsterdam: John Benjamins), 61–82.

73. Franklin, ‘Embodied Progress’.

74. Maggie Kirkman (2002), ‘Moving on, Living in the Subjunctive Mode: Revising Autobiographical Narratives after Infertility’, in The Fertile Imagination: Narratives of Reproduction, ed. Frances Thomson Salo, 59–82.

75. Gill and Lowes, ‘Gift Exchange’.

76. Scheper-Hughes, ‘The Tyranny’.

77. Papachristou et al., ‘Living Donor Liver’.

78. Zeiler, Guntram and Lennerling, 'Moral Tales'.

79. Sanner, Lagging, and Tibell, ‘The Kidney Recipient’s’.

80. However, as the interviewees told of their experiences it became clear that there have also been cases of friends becoming donors.

81. Individuals who – socially and perhaps also physically – have transitioned from female to male but have not had their uterus removed as part of sex-confirming surgery are potential donors. In such cases men, and also fathers, are also be potential donors.

82. Crombie and Franklin, ‘Family Issues’; Karrfelt, Berg, and Lindblad, ‘Renal Transplantation’; Lennerling et al., ‘Motives for Becoming’; Zeiler, Guntram and Lennerling, 'Moral Tales'.

83. Anna-Lena Wennberg et al. (2016), ‘Attitudes towards New Assisted Reproductive Technologies in Sweden: A Survey in Women 30–39 Years of Age’, *Acta Obstetricia et Gynecologica Scandinavica* 95, no. 1 (1 January 2016): 38–44, 10.1111/aogs.12781.

84. Liza Johannesson et al. (2018), ‘Living Uterus Donation and Transplantation: Experience of Interest and Screening in a Single Center in the United States’, *American Journal of Obstetrics and Gynecology* 218, no. 3 (1 March 2018): 331.e1-331.e7, 10.1016/j.ajog.2017.11.594.

85. Testa and Johannesson, ‘The Ethical’, 595.

86. Guntram, 'Hooked on'.

87. Solbrække and Bondevik, ‘Absent Organs—Present Selves’.

88. See also Allyse, ‘“Whole Again”’; Farrell, Flyckt, and Falcone, ‘The Call ’; Lotz, ‘Uterus Transplantation’; Mabel, Farrell, and Tzakis, ‘On Gender and Reproductive’; Guntram and Zeiler, 'The Ethics.

89. See also Horsburgh, ‘A Call’.

90. See also Kathy Davis (1995), Reshaping the Female Body: The Dilemma of Cosmetic Surgery (New York: Routledge); Kathy Davis (1997)s, Embodied Practices: Feminist Perspectives on the Body (London: Sage); Miriam Ulrich and Ann Weatherall (2000), ‘Motherhood and Infertility: Viewing Motherhood through the Lens of Infertility’, *Feminism & Psychology* 10, no. 3 (8 January 2000): 323–36, 10.1177/0959353500010003003.

91. Scheper-Hughes, ‘The Tyranny’.

92. See for example, Petrini et al., ‘Ethical Issues’.
